# Acute Myocardial Infarction Associated With Amphetamine Use and Smoking in a Young Healthy Individual

**DOI:** 10.7759/cureus.50323

**Published:** 2023-12-11

**Authors:** Ahmed A Alzeer, Ihab Suliman, Mohammed Altamimi, Abdullah M Alshudukhi, Abdulrahman A Alzeer, Eithar O Alwasidi

**Affiliations:** 1 Medical School, King Abdulaziz Medical City Riyadh, Riyadh, SAU; 2 Cardiology, Cardiac Center, King Abdulaziz Medical City, Riyadh, SAU; 3 College of Medicine, King Saud Bin Abdulaziz University for Health Sciences, Riyadh, SAU; 4 College of Medicine, Qassim University, Qassim, SAU

**Keywords:** st-elevation myocardial infarction (stemi), amphetamine, methamphetamine, substance-related disorders, sudden cardiac arrest, cardiac arrest, smoking, amphetamine-induced mi

## Abstract

One of the primary causes of death among methamphetamine users is cardiovascular disease, which is a result of the narrowing and spasm of blood vessels caused by the drug. This leads to increased blood pressure and heart rate, which can damage the heart muscle at the molecular level. The most common forms of chronic cardiovascular disease associated with methamphetamine use are coronary artery disease and cardiomyopathy.

Here, we report a case of myocardial infarction (MI) due to amphetamine use and smoking in a young healthy male who developed ST-elevation myocardial infarction, ventricular fibrillation (VF), and cardiac arrest. A 28-year-old male presented to the emergency department with chest pain and shortness of breath during exercise. Immediately upon presentation, electrocardiography was done which initially showed sinus tachycardia that progressed to right bundle branch block and ST elevation with a shark fin morphology, followed by VF and cardiac arrest. He was resuscitated and underwent percutaneous coronary intervention with stenting of the left anterior descending artery.

Cardiotoxic manifestations such as acute MI, heart failure, or arrhythmia related to misuse of amphetamines have been rarely documented. This case report describes the clinical course and management of a young male patient who suffered a life-threatening cardiac event triggered by smoking and amphetamine abuse.

## Introduction

Amphetamine use and smoking are common and harmful habits that can affect cardiovascular health [1,2]. According to the World Health Organization, amphetamine-type stimulants are the second most widely used illicit drugs in the world, and tobacco smoking is the leading cause of preventable death worldwide [3]. Both amphetamines and smoking can cause cardiovascular complications, such as myocardial infarction (MI), caused by accelerated atherosclerosis, rupture of preexisting atherosclerotic plaques, hypercoagulability, epicardial coronary artery spasm and increased myocardial oxygen demand, and prothrombotic effects [4,5]. MI due to amphetamine use and smoking at the same time is rare but potentially fatal and may present with atypical or absent symptoms [6]. Here, we report the case of a young male smoker and amphetamine user who upon arrival at the emergency department was momentarily stable but suddenly developed ST-elevation myocardial infarction (STEMI), ventricular fibrillation (VF), and cardiac arrest despite being young, healthy, and had no other risk factors. This case report describes the etiology, clinical presentation, diagnosis, management, and outcome of a young male patient who suffered a life-threatening cardiac event preceded by a significant history of smoking and amphetamine use while emphasizing the importance of prompt initiation of cardiopulmonary resuscitation (CPR), percutaneous coronary intervention (PCI), and medical management.

## Case presentation

A 28-year-old male had a long-standing history of heavy smoking (two packs per day for 14 years) and recreational amphetamine use. He also had uncontrolled bronchial asthma that caused him to have shortness of breath with minimal exertion. On the day of admission, he was performing his morning exercise at 6:20 a.m. when he developed severe chest pain, dyspnea, and vomiting. He was brought to the emergency department by his friend who witnessed the episode. At the time of arrival, he was alert and oriented to time and place, with no active complaints. His general appearance was comfortable, and he was hemodynamically stable on room air. His mental status was normal with no history of loss of consciousness. His cardiovascular examination revealed normal heart sounds, no carotid or femoral bruits on either side, normal and symmetrical peripheral pulses, and normal jugular venous pressure. His chest examination was unremarkable, with S1 and S2 heart sounds in regular rhythm with no murmurs or extra sounds. His lower limbs were well-perfused, with no edema. Upon further history taking, the patient revealed that he had recently used amphetamine, and a urine toxicology panel was positive for amphetamine.

**Figure 1:**
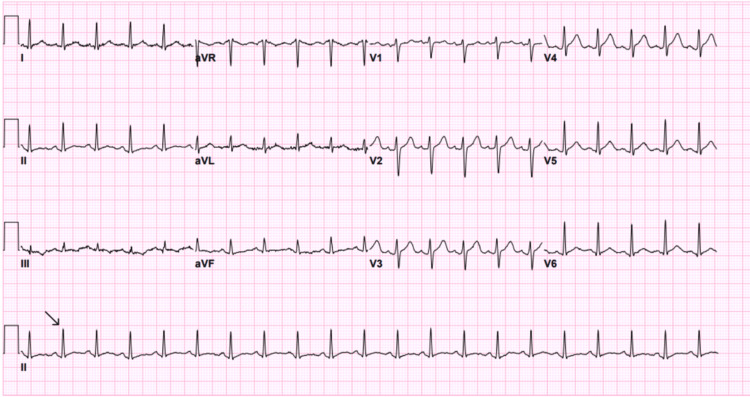
ECG showing sinus tachycardia of 136 beats per minute. Arrow indicating sinus tachycardia.

**Figure 2:**
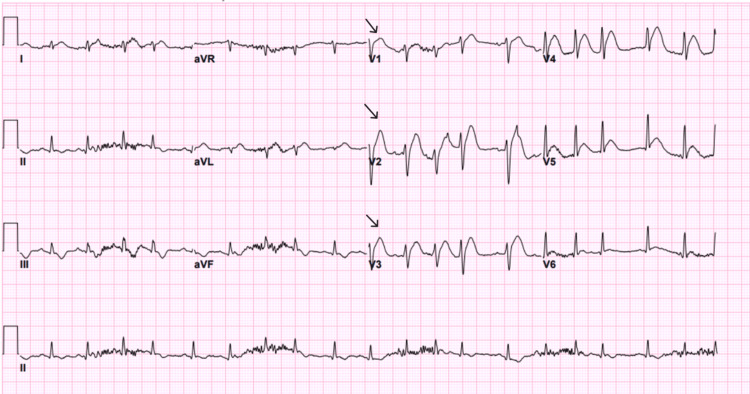
RBBB with STEMI in the anterior leads V1-V4. Arrows indicate the RSR pattern in V1-V3. RBBB: right bundle branch block; STEMI: ST-segment elevation myocardial infarction

**Figure 3:**
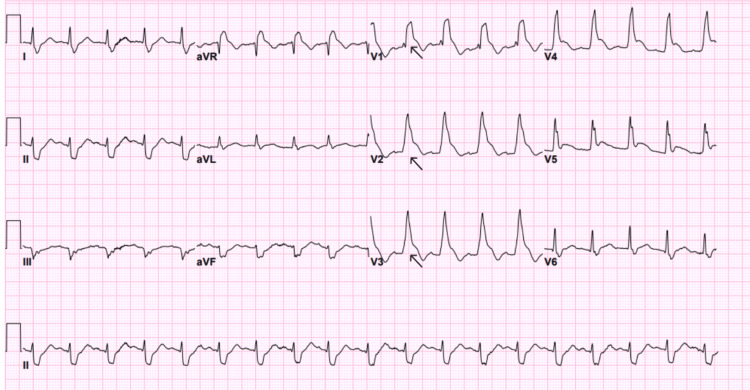
Shark fin ECG pattern. Arrow showing shark fin ECG pattern.

An electrocardiography (ECG) was done on the patient on arrival (Figure 1), which showed sinus tachycardia. However, after seven minutes of observation, he suddenly collapsed and became unresponsive. The patient was found to have cardiac arrest, and the cardiac monitor showed VF. Advanced cardiovascular life support (ACLS) protocol was started, and he was immediately resuscitated with three cycles of CPR. An automated external defibrillator (AED) delivered three shocks of 200 J, 200 J, and 250 J, as well as a single dose of amiodarone and epinephrine, which resulted in the return of spontaneous circulation. An ECG was done and showed a right bundle branch block with ST-segment elevation in the anterior leads, suggestive of acute anterior wall MI (Figure 2). Given the changes noted, the ECG was repeated, showing a progression to shark fin morphology (Figure 3). Cardiology was immediately contacted, and the catheterization laboratory was activated.

**Figure 4:**
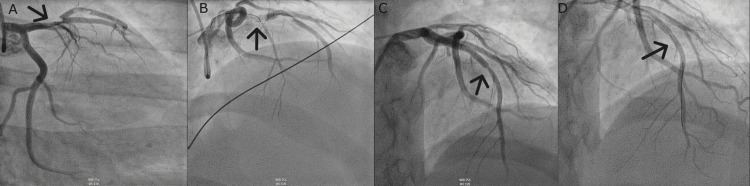
Urgent percutaneous coronary intervention stent placement. A: Large thrombus in the proximal segment of the left anterior descending artery and slow flow distally. B: Catheter inserted in the left anterior descending artery accessed through the right radial artery. C: Revascularization and the subsequent reperfusion of the left anterior descending artery with stenosis. D: Stent placement and resolution of stenosis.

He was instantly given aspirin, clopidogrel, heparin, and nitroglycerin and transferred to the catheterization laboratory for urgent PCI. Overall, 2 mL of 1% xylocaine was used to infiltrate the right radial area for local anesthetic effect. The right radial artery was accessed using the modified Seldinger technique and a 6-French sheath was inserted over a guidewire. A heparin bolus was administered. A 6-French XB 3.0 guiding catheter was inserted using fluoroscopic guidance over a guidewire and selective angiography of the left coronary artery was performed. A 190 cm .014 in BMW wire was used to cross the left anterior descending artery (LAD) lesion under fluoroscopic guidance. A 190 cm .014 in BMW wire was used to cross the diagonal lesion under fluoroscopic guidance. A 4 × 38 mm Resolute Onyx drug-eluting stent was deployed at the proximal LAD at 10 ATM for 16 seconds (Figure 4A). A 3.50 mm × 20 mm Raiden non-compliant balloon catheter was used to post-dilate the proximal LAD stent at 14 ATM for eight seconds (Figure 4B). A second inflation was done to post-dilate the proximal LAD stent up to 20 ATM. A 6-French Export catheter was used to aspirate the clot from LAD (Figure 4C). A 5-French JR 3.5 diagnostic catheter was inserted using fluoroscopic guidance over a guidewire, and selective angiography of the right coronary artery was performed (Figure 4D). A TR Band was applied and inflated over the right radial artery (Figure 4).

**Figure 5:**
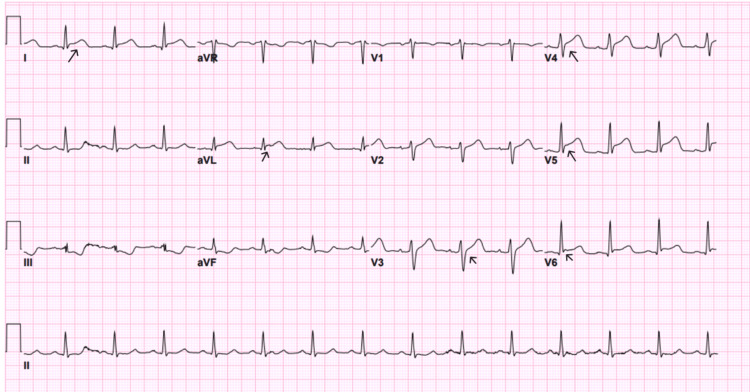
Day zero post-percutaneous coronary intervention ECG.

After PCI, ECG showed an ST-segment elevation in leads I, aVL, and V3-6 (Figure 5).

**Figure 6:**
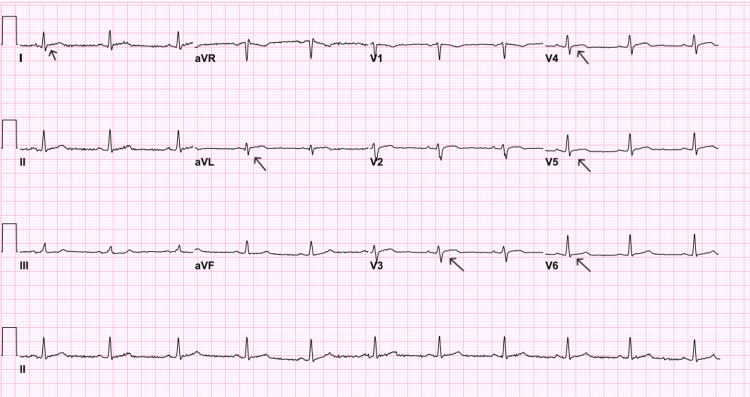
ECG before discharge.

He was then admitted to the coronary care unit for further management and monitoring. Over the next five days, a serial ECG was done, which showed normalization of his previous ST-segment elevation in leads I, aVL, and V3-6 (Figure 6).

**Figure 7:**
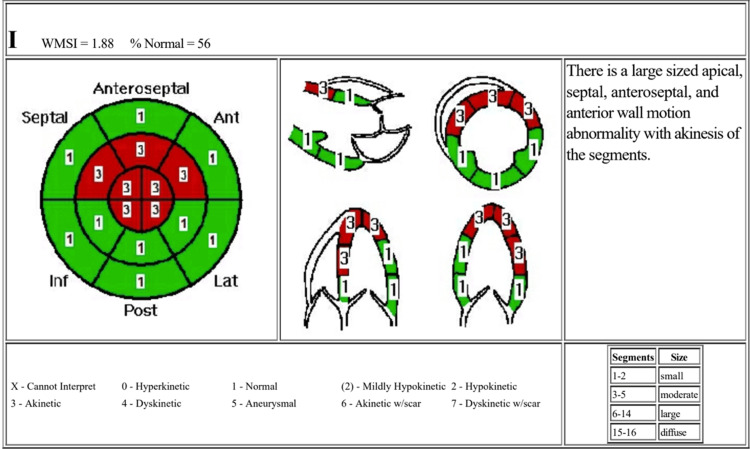
Echocardiogram after percutaneous coronary intervention. The left ventricle is normal in size and wall thickness with mild to moderately reduced systolic function. Ejection fraction of 45-50%. The transmitral spectral Doppler flow pattern is normal for age. There are regional wall motion abnormalities as specified.

He recovered well from the procedure and was discharged after five days with appropriate medication and lifestyle counseling. He underwent an echocardiogram to assess his cardiac function during his stay, which was unremarkable, except for some left ventricular wall motion abnormalities (Figure 7). He was advised to quit smoking and amphetamine use and to follow up with his primary care physician and cardiologist regularly. Unfortunately, the patient missed his follow-up appointments, which made his long-term recovery unclear.

## Discussion

A common complaint that is correlated with amphetamine use is chest pain, which accounts for 28% of all admissions to the hospital that are associated with amphetamine. This presentation of chest pain is mainly linked with the side effects of amphetamine such as hypertension and tachycardia, yet 25% of these cases are attributed to acute coronary syndrome (ACS) and 8% were more serious cardiovascular complications [5].

The majority of acute myocardial infarction (AMI) occurs in patients with commonly known risk factors that cause atherosclerosis, plaque rupture, coronary vasoconstriction, increased myocardial oxygen demand, platelet activation, and thrombosis [7,8]. However, there is still a percentage of patients who have less common risk factors such as amphetamine use that end up with AMI.

The management of amphetamine-induced MI is similar to that of other causes of MI, except avoiding beta-blockers in the acute phase due to the risk of unopposed alpha-adrenergic stimulation and worsening hypertension [9] The use of vasodilators such as nitroglycerin and calcium channel blockers may be beneficial in relieving coronary spasm [10,11]. Similar to other causes of MI, the long-term prognosis of amphetamine-induced MI depends on the extent of myocardial damage, the presence of other comorbidities, and the cessation of amphetamine use and smoking [12].

This patient’s presentation was unusual because he initially experienced typical ACS symptoms such as chest pain, shortness of breath, and diaphoresis before presenting to the hospital. However, in the emergency department, the patient did not have any signs that would indicate amphetamine abuse or ACS, and he was clinically and vitally stable. This intermittent period of stability lasted seven minutes, followed by unresponsiveness and hemodynamic instability. Prompt ECG was done, and VF and cardiac arrest were found, which required immediate intervention. He was fortunate to receive timely CPR, AED, and PCI, which saved his life and preserved his cardiac function.*

## Conclusions

The rapid and unpredictable progression of ACS to AMI, VF, and cardiac arrest necessitates swift identification and management. Our case demonstrated that patients who have a history of amphetamine use and smoking can present in an atypical fashion of momentary clinical and hemodynamic stability, which is preceded by normal ACS presentation and followed by imminent cardiopulmonary collapse.
